# Are quantitative features of lung nodules reproducible at different CT acquisition and reconstruction parameters?

**DOI:** 10.1371/journal.pone.0240184

**Published:** 2020-10-15

**Authors:** Barbaros S. Erdal, Mutlu Demirer, Kevin J. Little, Chiemezie C. Amadi, Gehan F. M. Ibrahim, Thomas P. O’Donnell, Rainer Grimmer, Vikash Gupta, Luciano M. Prevedello, Richard D. White

**Affiliations:** 1 Department of Radiology, The Ohio State University College of Medicine, Columbus, Ohio, United States of America; 2 Siemens Healthineers, Malvern, Pennsylvania, United States of America and Erlangen, Germany; INSERM, FRANCE

## Abstract

Consistency and duplicability in Computed Tomography (CT) output is essential to quantitative imaging for lung cancer detection and monitoring. This study of CT-detected lung nodules investigated the reproducibility of volume-, density-, and texture-based features (outcome variables) over routine ranges of radiation dose, reconstruction kernel, and slice thickness. CT raw data of 23 nodules were reconstructed using 320 acquisition/reconstruction conditions (combinations of 4 doses, 10 kernels, and 8 thicknesses). Scans at 12.5%, 25%, and 50% of protocol dose were simulated; reduced-dose and full-dose data were reconstructed using conventional filtered back-projection and iterative-reconstruction kernels at a range of thicknesses (0.6–5.0 mm). Full-dose/B50f kernel reconstructions underwent expert segmentation for reference Region-Of-Interest (ROI) and nodule volume per thickness; each ROI was applied to 40 corresponding images (combinations of 4 doses and 10 kernels). Typical texture analysis metrics (including 5 histogram features, 13 Gray Level Co-occurrence Matrix, 5 Run Length Matrix, 2 Neighboring Gray-Level Dependence Matrix, and 3 Neighborhood Gray-Tone Difference Matrix) were computed per ROI. Reconstruction conditions resulting in no significant change in volume, density, or texture metrics were identified as “compatible pairs” for a given outcome variable. Our results indicate that as thickness increases, volumetric reproducibility decreases, while reproducibility of histogram- and texture-based features across different acquisition and reconstruction parameters improves. To achieve concomitant reproducibility of volumetric and radiomic results across studies, balanced standardization of the imaging acquisition parameters is required.

## Introduction

Lung nodules have traditionally been evaluated with two-dimensional (2-D) linear measurements on chest Computed Tomography (CT) (e.g., Response Evaluation Criteria in Solid Tumors (RECIST) [1]). However, three-dimensional (3-D) volumetric assessments of lung nodules are gaining importance due to: 1. improved representation of disease extent and therapeutic-response; and 2. less user-dependency and higher reproducibility of the results [2]. Concurrently, new diagnostic and treatment paradigms increasingly emphasize the value of quantitative radiomic features of lung nodules and surrounding lung tissue as indicators of tumor type, aggressiveness, and/or responsiveness to treatment [3, 4]. However, the associated quantitative metrics assume adequacy and uniformity in CT data acquisition and reconstruction despite well-recognized wide inter-scan variability in: 1. examination protocoling for image acquisition; 2. image reconstructions and displays; and 3. CT scanning capabilities and performances. Although the susceptibility of several lung nodule measurements (volumetric or radiomic) to variations in individual CT acquisition parameters has already been recognized based on preliminary studies of clinical or phantom data [5–8], the concomitant effects that ranges of radiation-dose, reconstruction kernel, and slice thickness have on nodule volume and texture features have not yet been fully investigated.

The purpose of this research was to: 1. determine the impact of a variety of imaging acquisition and reconstruction parameters on lung nodule volumes and radiomic features; 2. identify potential imaging acquisition parameters that allow consistency and reproducibility of volumetric and/or radiomic features of lung nodules.

## Materials and methods

This work was approved by The Ohio State University Institutional Review Board (2015H0185, 2017H0100). This retrospective study used de-identified images, and consent was not obtained.

### Acquisition and reconstruction parameter variations

Raw Digital Imaging and Communications in Medicine (DICOM) data from 23 non-contrast-enhanced chest CT examinations demonstrating a lung nodule were acquired from either single-source (Definition AS, AS Plus, Edge) or dual-source (Definition Flash) multi-detector CT systems [Somatom series: Siemens Healthineers, Forchheim, Germany (https://www.healthcare.siemens.com/computed-tomography)] with ranges of settings (Tube voltage = 100–140 kV, Q.ref.mAs = 50–100 mAs, Eff.mAs = 41–154 mAs, and pitch = 0.6–1.0). Based on this data, chest CT data sets underwent the following:

1. Simulation at different dose levels (simulated 12.5%, 25%, 50%) using a previously validated simulation tool [9–11], as well as reconstruction at 100% of total dose of clinical protocols [ReconCT: Siemens Healthineers, Forchheim, Germany].
2. Reproduction with 10 different kernels based on either Filtered Back Projection (FBP) or Iterative Reconstruction (IR) (FBP: B31f, B40f, B50f, B60f, B70f; SAFIRE (strength 3): I26f, I31f, I40f, I50f, I70f) [ReconCT: Siemens Healthineers, Forchheim, Germany].
3. Recreation at 8 different slice thicknesses (0.6, 0.75, 1.0, 1.5, 2.0, 3.0, 4.0, and 5.0 mm) using an offline reconstruction system [ReconCT: Siemens Healthineers, Forchheim, Germany].

Based on combinations of the aforementioned variations in acquisition or reconstruction parameters, 320 versions of each examination (i.e., 4 doses x 10 kernels x 8 thicknesses) were created.

### Segmentation and volume measurement

**Fig 1:**
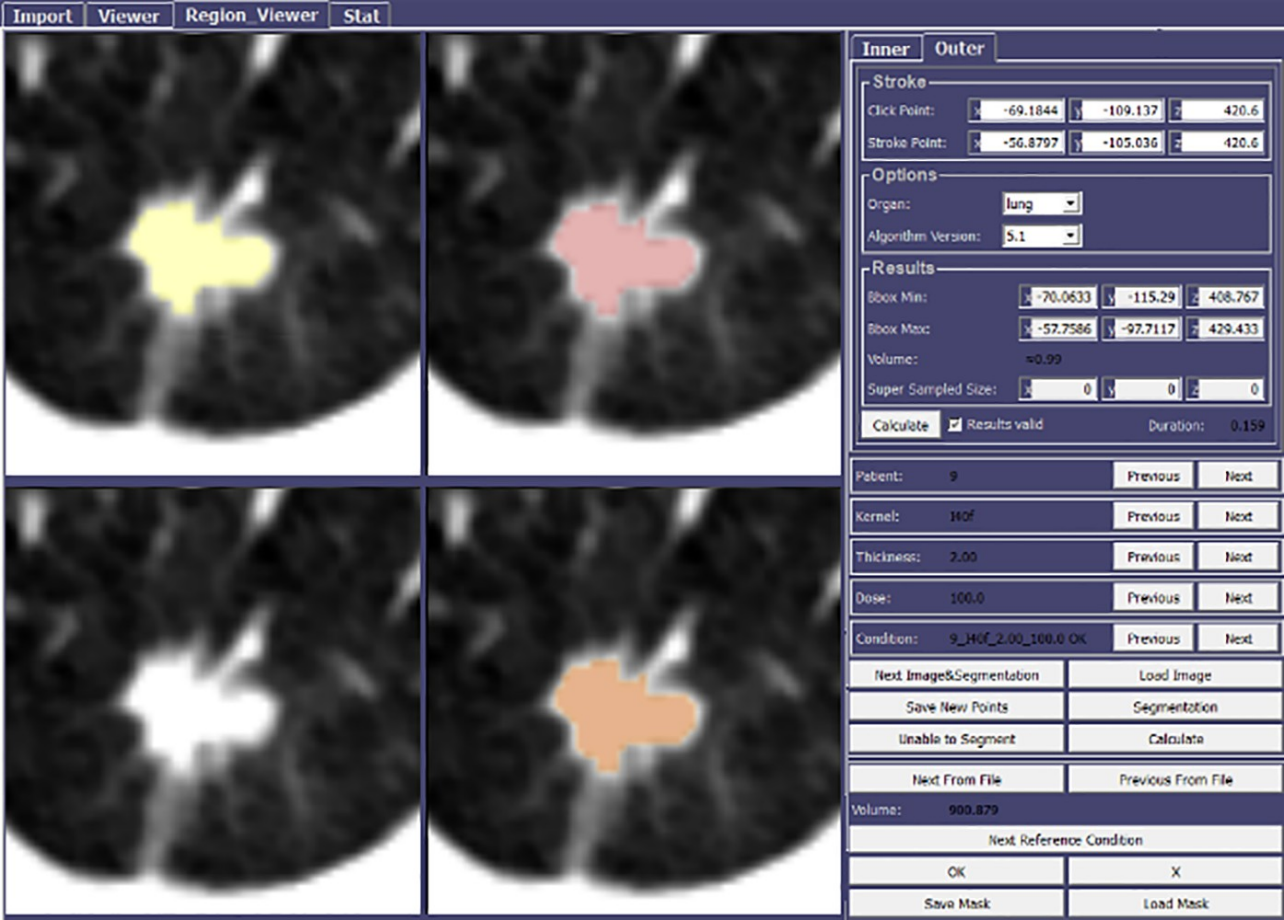
A custom GUI allowed thoracic radiologists to evaluate nodules. For the purposes of this study, only the solid components were considered. Semi-automated segmentations made by radiologists for a given nodule are shown. Top left in Yellow: Radiologist 1; top right in Red: Radiologist 2; bottom left: non-segmented original; bottom right in Orange, both radiologist’s segmentations combined.

In order to display the nodules to dedicated thoracic radiologists (CCA and GFMI each with 6–8 years of post-fellowship experience), a custom Graphical User Interface (GUI) [12] was built [MeVisLab version 2.8, Windows 64 bit, VS2013: Bremen, Germany (https://www.mevislab.de/)] (Fig 1). This GUI integrated the functionalities of a commercial nodule segmentation algorithm [syngo.via MM Oncology: Siemens Healthineers, Forchheim, Germany (https://www.siemens-healthineers.com/medical-imaging-it/syngoviaspecialtopics/syngo-via-for-oncology)] as they would appear within an established commercial post-processing platform [syngo.via: Siemens Healthineers, Forchheim, Germany (https://www.healthcare.siemens.com/medical-imaging-it/advanced-visualization-solutions/syngovia)].

**Fig 2:**
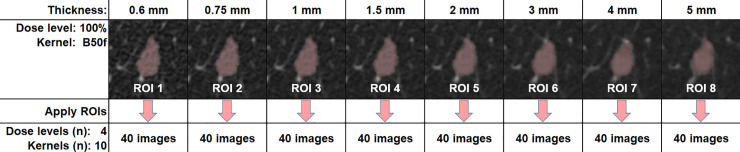
Segmentation procedure for lung nodules. Each nodule was segmented from 100%-dose/FBP B50f-kernel images reconstructed at 8 different slice thicknesses. The resulting 8 thickness-specific ROI stacks were then applied to the corresponding images reconstructed at 4 different dose levels and 10 different kernels at stable slice thickness.

For each chest CT examination, sets of 100%-dose/FBP B50f-kernel images were reconstructed at the aforementioned 8 different slice thicknesses, with nodule extent and shape previously independently confirmed by consensus between the two dedicated thoracic radiologists. This resulted in 8 reference Region-Of-Interest (ROI) stacks, each corresponding to a specific slice thickness, which were then applied to the 40 different dose-kernel combinations (i.e., 4 doses x 10 kernels) at constant slice thickness (Fig 2).

### Analysis of reproducibility of volumetric measurements

**Fig 3:**
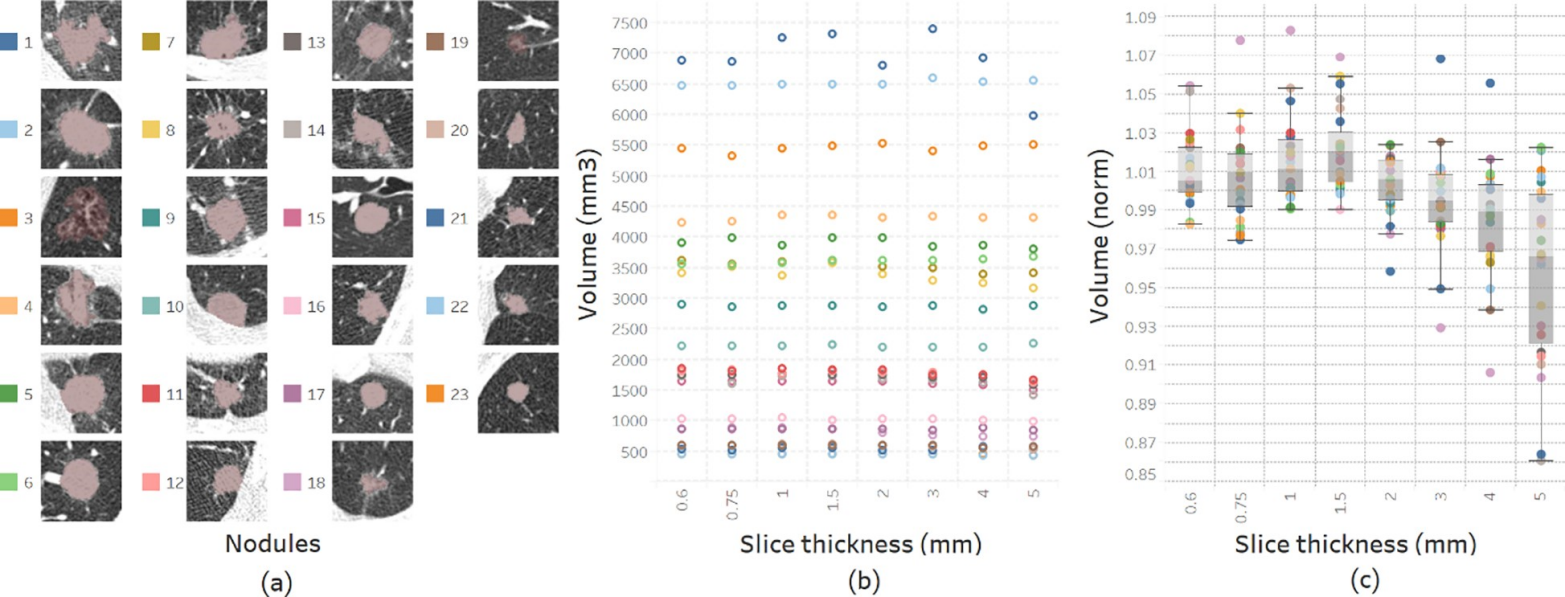
Methodologies used during volumetric assessment of lung nodules. The following represent expert segmentation of lung nodules: (a) Reference radiologist-segmented ROI stacks obtained using 100%-dose level, a FBP B50f kernel, and 1-mm slice thickness. Nodules are sorted by their average volumes and numbered 1 to 23. (b) Calculated volumes for each nodule using 8 reference ROIs depending on slice thicknesses (colors indicate the corresponding nodules shown on the left). (c) Normalized volumes of nodules obtained using their average volumes.

Using automatic 3-D segmentation, 8 volumetric measurements were obtained for each nodule (Fig 3A) corresponding to each slice thickness (Fig 3B) and normalized by their averages using (1) as follows (*V*_*i*_: nodule volume for i-th thickness):

$$V\left(norm\right)=\frac{V_{i}\left(mm^{3}\right)}{\frac{1}{8}\sum_{k=1}^{8}V_{k}\left(mm^{3}\right)}$$ (1)

Using the distributions of normalized volumes for each slice thickness (Fig 3C), t statistics were calculated using 2-tailed t-test (2) to evaluate the compatibilities of slice thicknesses based on volumetric measurements. If t<1.96 (P<0.05), slice thicknesses are accepted as compatible with 95% confidence interval; *m*_1_, *m*_2_: means; *s*_1_, *s*_2_: standard deviations; *n*_1_, *n*_2_: number of samples (nodules):

$$t=\frac{abs(m_{1}-m_{2})}{\sqrt{\frac{{\left(s_{1}\right)}^{2}}{n_{1}}+\frac{{\left(s_{2}\right)}^{2}}{n_{2}}}}$$ (2)

### Image-feature extraction

A range of extracted radiomic image features was assessed (Table 1). They included 28 image-texture features (available in MeVisLab) as follows: 1. 5 Histogram-based features; 2. 13 Gray Level Co-occurrence Matrix (GLCM)-based features; 3. 5 Run Length Matrix (RLM)-based features; 4. 2 Neighboring Gray-Level Dependence Matrix (NGLDM)-based features, and 5. 3 Neighborhood Gray-Tone Difference Matrix (NGTDM) based features which were computed for all 320 segmented image volumes of each nodule.

**Table 1:** Extracted features.

Group	Feature	Definition
I. Histogram	1. Mean	$\bar{X}=\frac{1}{N}\sum_{i=1}^{N}X(i)$
	2. Contrast	$\sqrt{\frac{1}{N}\sum_{i=1}^{N}{\left(X(i)-\bar{X}\right)}^{2}}$
	3. Standard deviation	$\sqrt{\frac{1}{N-1}\sum_{i=1}^{N}{\left(X(i)-\bar{X}\right)}^{2}}$
	4. Skewness	$\frac{\frac{1}{N}\sum_{i=1}^{N}{\left(X(i)-\bar{X}\right)}^{3}}{{\left(\sqrt{\frac{1}{N-1}\sum_{i=1}^{N}{\left(X(i)-\bar{X}\right)}^{2}}\right)}^{3}}$
	5. Kurtosis	$\frac{\frac{1}{N}\sum_{i=1}^{N}{\left(X(i)-\bar{X}\right)}^{4}}{{\left(\sqrt{\frac{1}{N}\sum_{i=1}^{N}{\left(X(i)-\bar{X}\right)}^{2}}\right)}^{2}}$
II. GLCM	6. Homogeneity (asm)	∑_*i*_∑_*j*_{*p*(*i*,*j*)}^2^
	7. Contrast	$\sum_{n=0}^{N_{g}-1}n^{2}\{\sum_{i=1}^{N_{g}}\sum_{j=1}^{N_{g}}p(i,j)\}$
	8. Correlation	$\frac{\sum_{i}\sum_{j}(ij)p(i,j)-\mu_{x}\mu_{y}}{\sigma_{x}\sigma_{y}}$
	9. Variance	∑_*i*_∑_*j*_(*i*−μ)^2^*p*(*i*,*j*)
	10. Inverse difference moment	$\sum_{i}\sum_{j}\frac{1}{1+{\left(1-j\right)}^{2}}p(i,j)$
	11. Sum average	$\sum_{i=2}^{2N_{g}}ip_{x+y}(i)$
	12. Sum entropy	$-\sum_{i=2}^{2N_{g}}p_{x+y}(i)\log\left\{p_{x+y}(i)\right\}$
	13. Sum variance	$\sum_{i=2}^{2N_{g}}{\left(i-\mu_{x+y}\right)}^{2}p_{x+y}(i)$
	14. Entropy	$-\sum_{i}\sum_{j}p(i,j)\log\left(p(i,j)\right)$
	15. Difference variance	$\sum_{i=0}^{N_{g}-1}{\left(i-\mu_{x-y}\right)}^{2}p_{x-y}(i)$
	16. Difference entropy	$-\sum_{i=0}^{N_{g}-1}p_{x-y}(i)\log\left\{p_{x-y}(i)\right\}$
	17. Measures of correlation1	$\frac{Ent-HXY1}{max\{HX,HY\}}$
	18. Measures of correlation2	$\sqrt[{2}]{1-e^{-2(HXY2-Ent)}}$
III. RLM	19. Short run emphasis	$\frac{\sum_{i=1}^{N_{g}}\sum_{j=1}^{N_{r}}\left[\frac{p(i,j|\theta )}{j^{2}}\right]}{\sum_{i=1}^{N_{g}}\sum_{j=1}^{N_{r}}p(i,j|\theta )}$
	20. Long run emphasis	$\frac{\sum_{i=1}^{N_{g}}\sum_{j=1}^{N_{r}}j^{2}p(i,j|\theta )}{\sum_{i=1}^{N_{g}}\sum_{j=1}^{N_{r}}p(i,j|\theta )}$
	21. Grey level non-uniformity	$\frac{\sum_{i=1}^{N_{g}}{\left[\sum_{j=1}^{N_{r}}p(i,j|\theta )\right]}^{2}}{\sum_{i=1}^{N_{g}}\sum_{j=1}^{N_{r}}p(i,j|\theta )}$
	22. Run length non-uniformity	$\frac{\sum_{j=1}^{N_{r}}{\left[\sum_{i=1}^{N_{g}}p(i,j|\theta )\right]}^{2}}{\sum_{i=1}^{N_{g}}\sum_{j=1}^{N_{r}}p(i,j|\theta )}$
	23. Run percentage	$\sum_{i=1}^{N_{g}}\sum_{j=1}^{N_{r}}\frac{p(i,j|\theta )}{N_{p}}$
IV. NGLDM	24. Small Number Emphasis	$\sum_{k=1}^{K}\sum_{s=1}^{S}\left[Q(k,s)/s^{2}\right]/\sum_{k=1}^{K}\sum_{s=1}^{S}Q(k,s)$
	25. Large Number Emphasis	$\sum_{k=1}^{K}\sum_{s=1}^{S}\left[s^{2}Q(k,s)\right]/\sum_{k=1}^{K}\sum_{s=1}^{S}Q(k,s)$
V. NGTDM	26. Coarseness	${\left[\sum_{i=0}^{L_{h}}p_{i}s(i)\right]}^{-1}$
	27. Complexity	$\sum_{i=0}^{L_{h}}\sum_{j=0}^{L_{h}}\{(|i-j|)/\left(n^{2}\left(p_{i}+p_{j}\right))\right\}\left\{p_{i}s(i)+p_{j}s(j)\right\}$
	28. Texture Strength	$\frac{\left[\sum_{i=0}^{L_{h}}\sum_{j=0}^{L_{h}}\left(p_{i}+p_{j}\right){\left(i-j\right)}^{2}\right]}{\left[\sum_{i=0}^{L_{h}}s(i)\right]}$

### Compatibility of image reconstruction conditions based on radiomic features

Means and standard deviations of the aforementioned 28 image features were computed for each of the 320 segmented images of each nodule. Statistically significant changes in image features were evaluated using 2-tailed t-test. Reconstruction condition pairs that satisfy *t* values of lower than <1.96 (P<0.05) were accepted as compatible with 95% confidence interval. Compatibility ratios were calculated using Eq 3; ${CR}_{{(R}_{i},R_{j})}$: compatibility ratio of reconstruction condition pair (*R*_*i*_,*R*_*j*_); *R*_*i*_: *i*-th reconstruction condition (*i* = 1,2,…,320); $C_{(R_{i},R_{j)}}(f,p)$: compatibility of (*R*_*i*_,*R*_*j*_) pair for feature *f* (1,…,28) and patient *p* (1,…,23) calculated using t-test (Eq 2):

$${CR}_{{(R}_{i},R_{j})}=\frac{\sum_{f=1}^{28}\sum_{p=1}^{23}C_{(R_{i},R_{j)}}(f,p)}{28x23}x100$$ (3)

**Fig 4:**
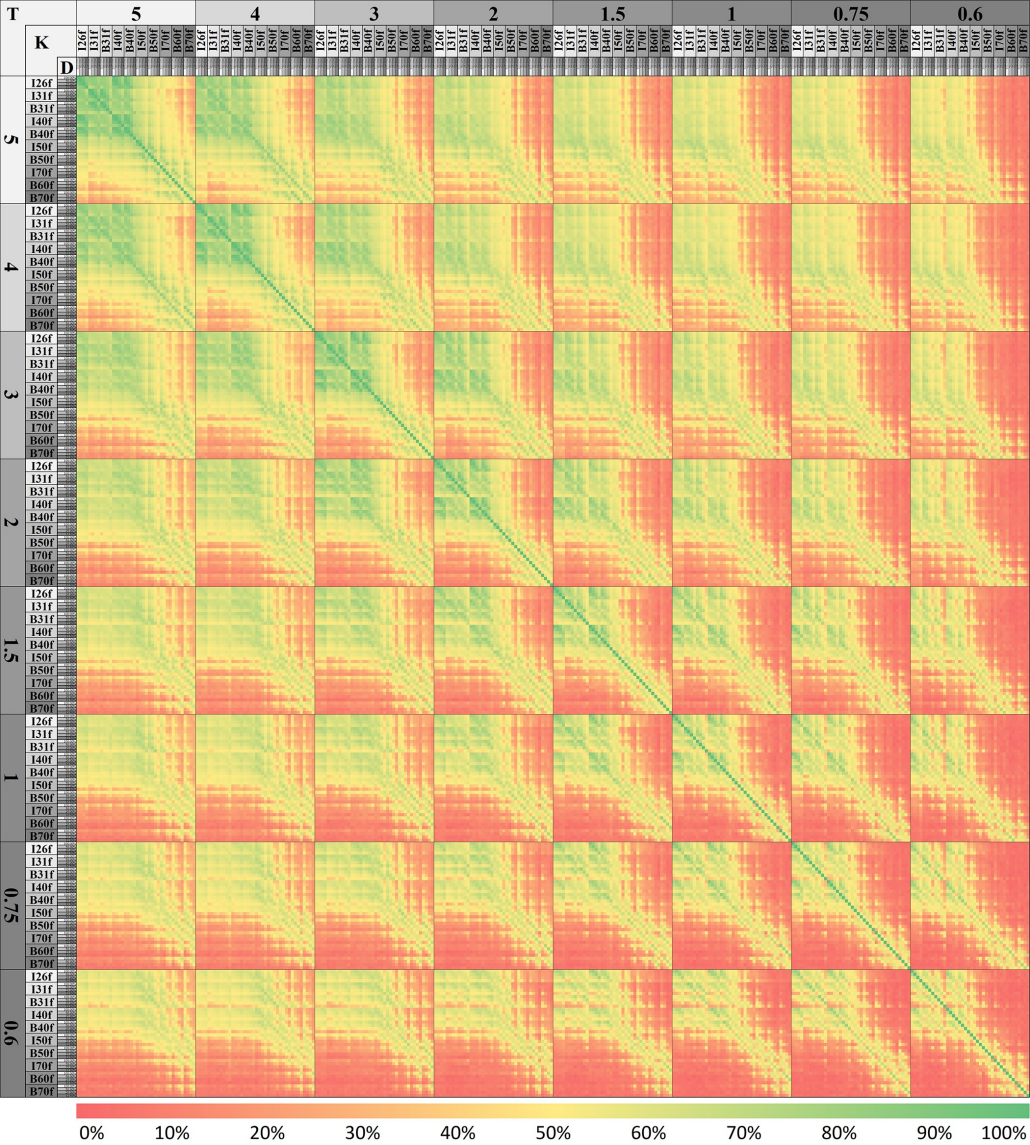
Reconstruction-condition compatibility map based on extracted features and patients. Intersections of conditions are highlighted (Red: incompatible, Green: compatible) based on their compatibility ratios calculated using t-test. Diagonal shows 100% compatibility which satisfies all comparisons (28x23). Changing reconstruction parameters (thickness/dose/kernel-sharpness) decreases the compatibility. In order to obtain higher compatibility, changes to the reconstruction parameters should be applied carefully. For example if thickness needs to be switched from 2 mm to 0.75 mm, softer kernels and/or higher dose levels are needed as seen from the intersection of two thicknesses. Data are available in S1 Data.

We produced a compatibility map of reconstruction conditions (Fig 4) to highlight effects of changes in slice thickness (T), kernel sharpness (K), and dose (D). Reconstruction condition parameters are sorted based on total number of compatibilities. Thickness order: 5mm, 4mm, 3mm, 2mm, 1.5mm, 1mm, 0.75mm, 0.6mm. Kernel order: I26f, I31f, B31f, I40f, B40f, I50f, B50f, I70f, B60f, B70f. Dose level order: 100%, 50%, 25%, 12.5%.

## Results

### Reproducibility of volumetric measurements

**Fig 5:**
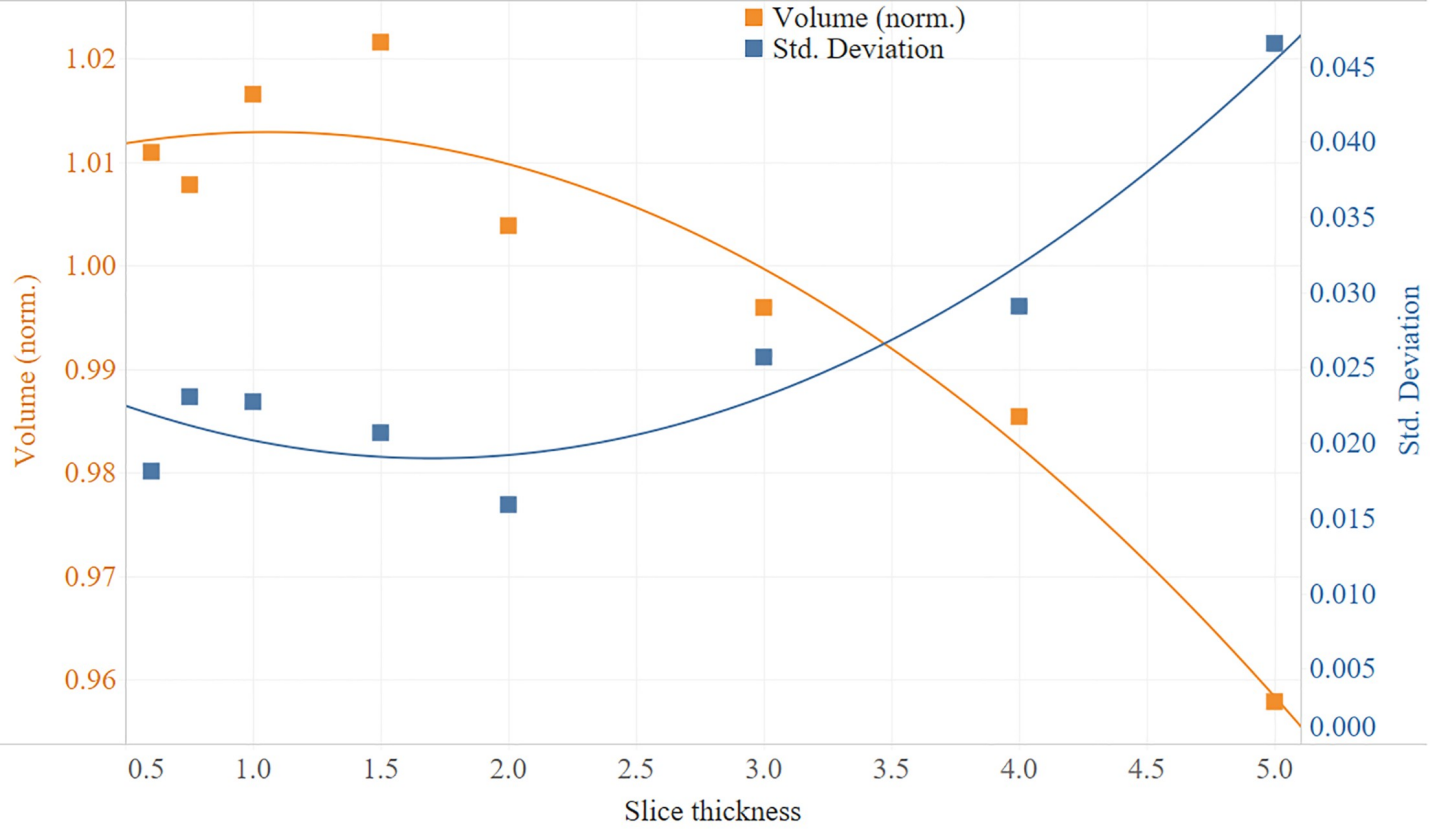
Normalized volumetric measurements and trend lines based on slice thickness.

**Fig 6:**
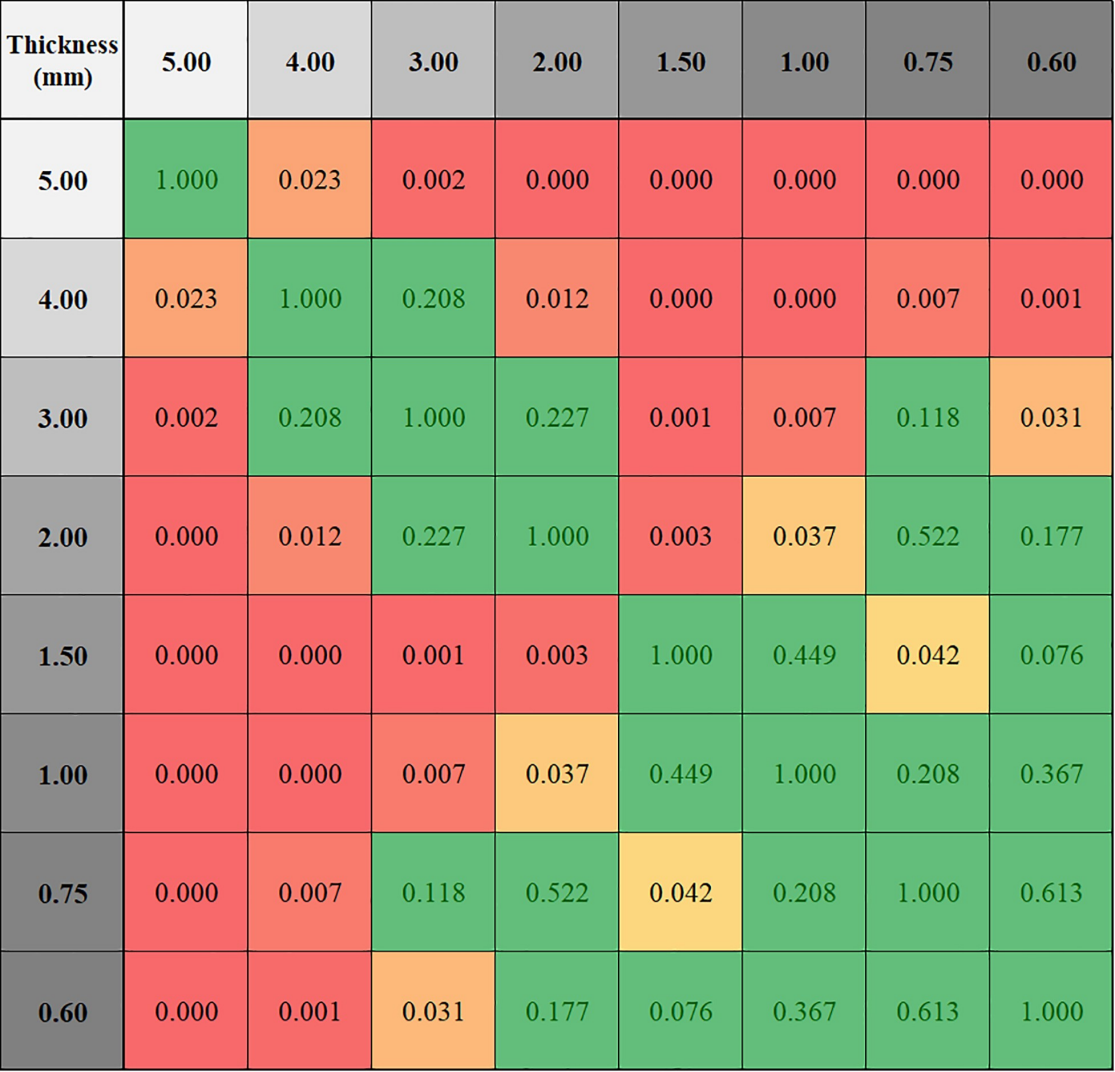
P values for compatibility analysis of slice thicknesses based on volumetric measurements. Higher P value indicates higher compatibility. Intersection of compatible thicknesses with P values higher than 0.05 are highlighted with green; P values lower than 0.05 (highlighted with orange to red) indicate incompatible slice thicknesses.

Volume comparisons of the segmented nodules were performed on 8 slice thicknesses on all 23 cases (Fig 5). The slice thickness resulting in the least amount of volumetric variability was 2mm, with +0.39%±1.59 (mean±SD) variation from average volume. On the other hand, 1.5-mm thickness gave the highest volumes with +2.16%±2.07, and 5-mm the lowest volumes with -4.22%±4.65. While standard deviations were relatively stable for slice thicknesses below 2mm, increasing thickness beyond 2mm was associated with rapidly increasing standard deviation. The assessment of volumetric reproducibility shows that increasing slice thickness decreases compatibility (Fig 6).

### Reproducibility of radiomic features

**Fig 7:**
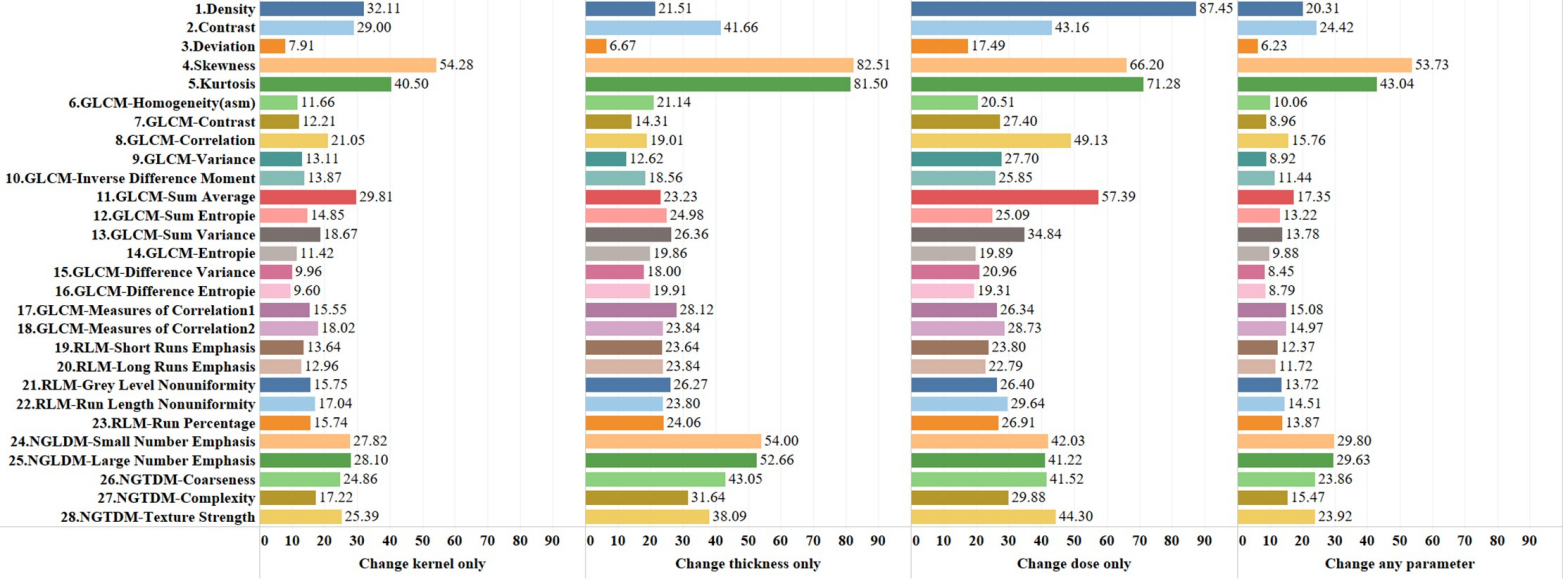
Percentage compatibilities of features based on dose, kernel, and thickness changes.

**Fig 8:**
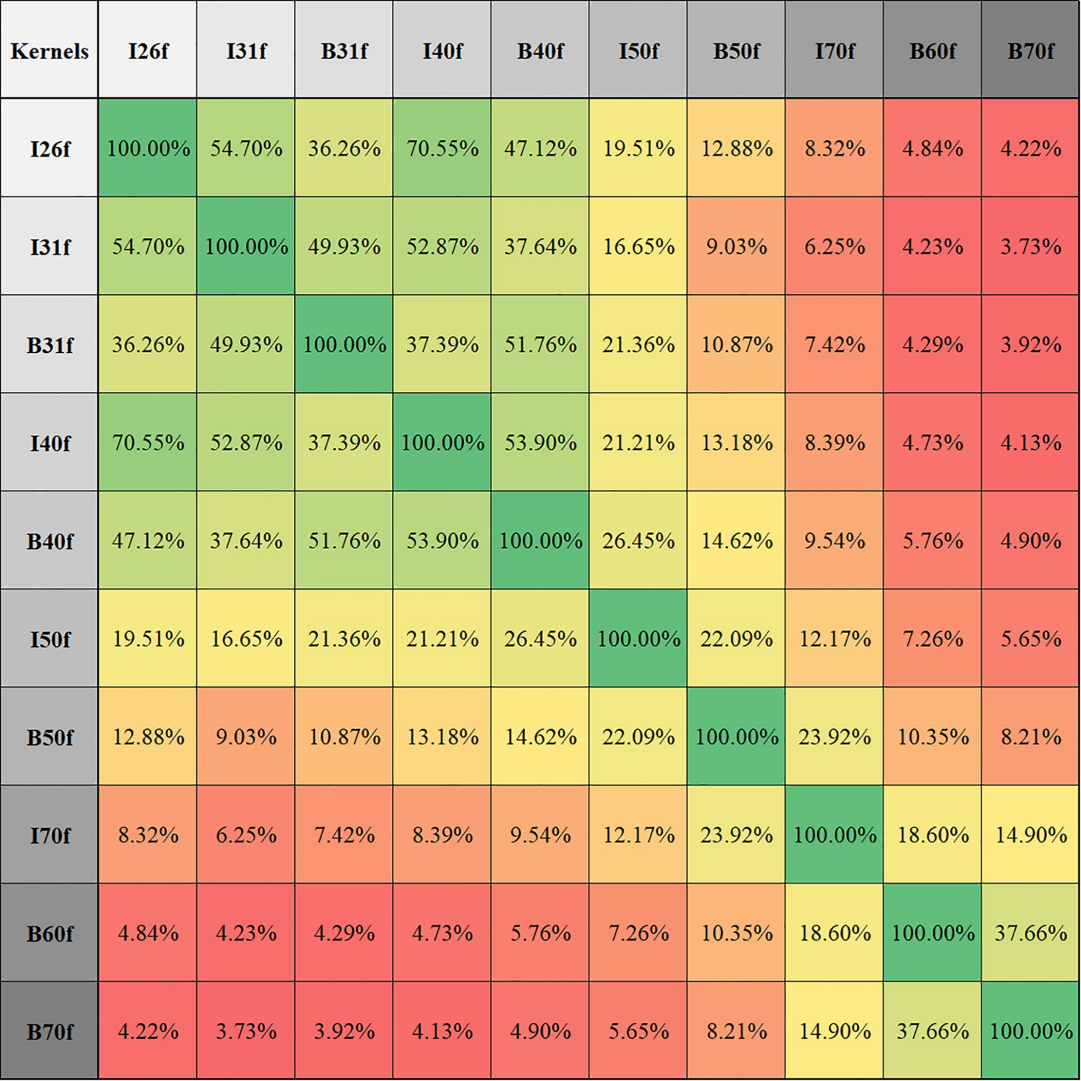
Percentage of compatible texture features in different kernel pairs while keeping slice thickness and dose levels fixed for all 23 patients and 28 features. For example, by changing the kernel from I31f to I26f, on average, 54.7% of the 28 texture features will be statistically the same (compatible) in our patient population under the same slice thickness and dose level configurations (32 different conditions).

**Fig 9:**
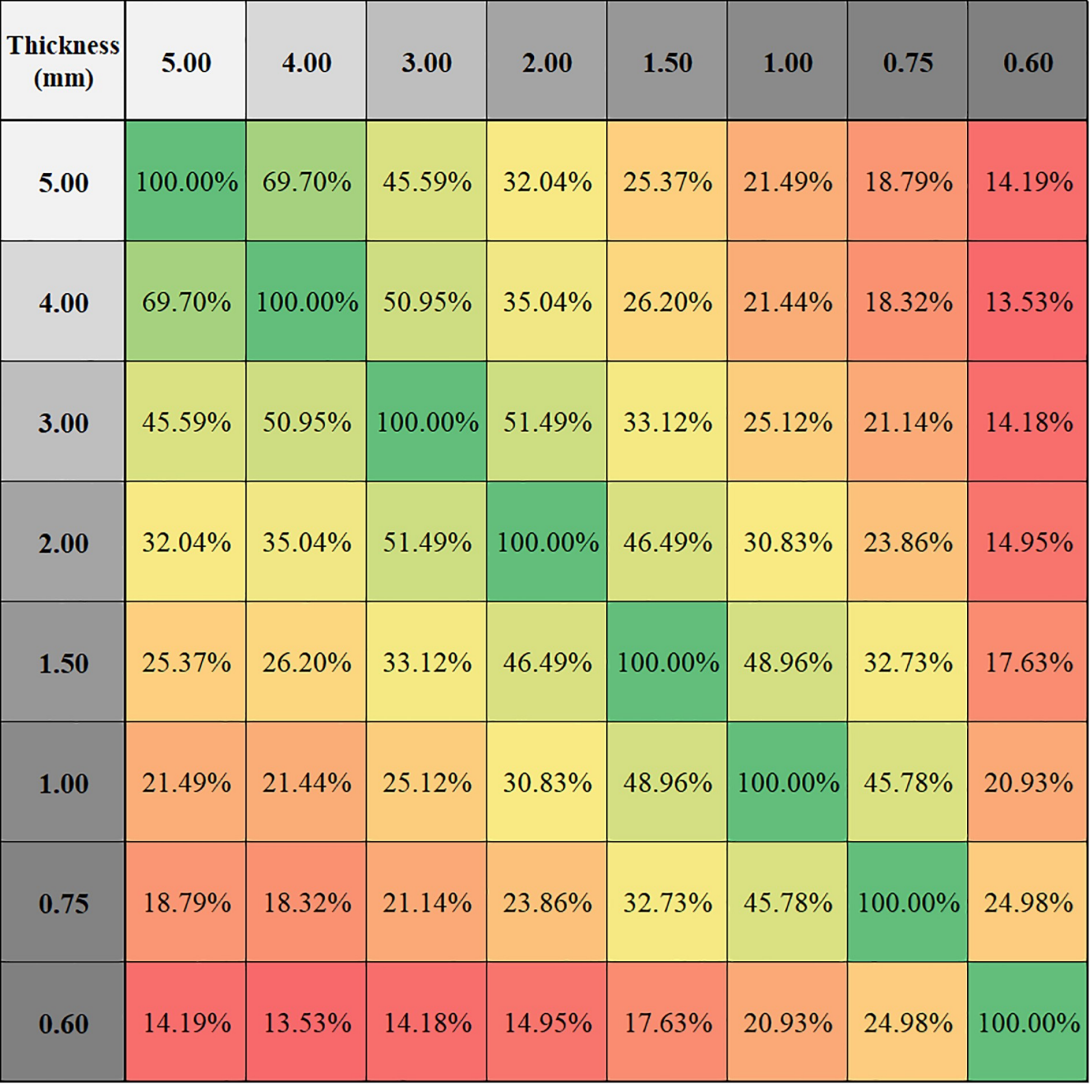
Percentage of compatible texture features in different slice thickness pairs while keeping kernel and dose levels fixed for all 23 patients.

**Fig 10:**
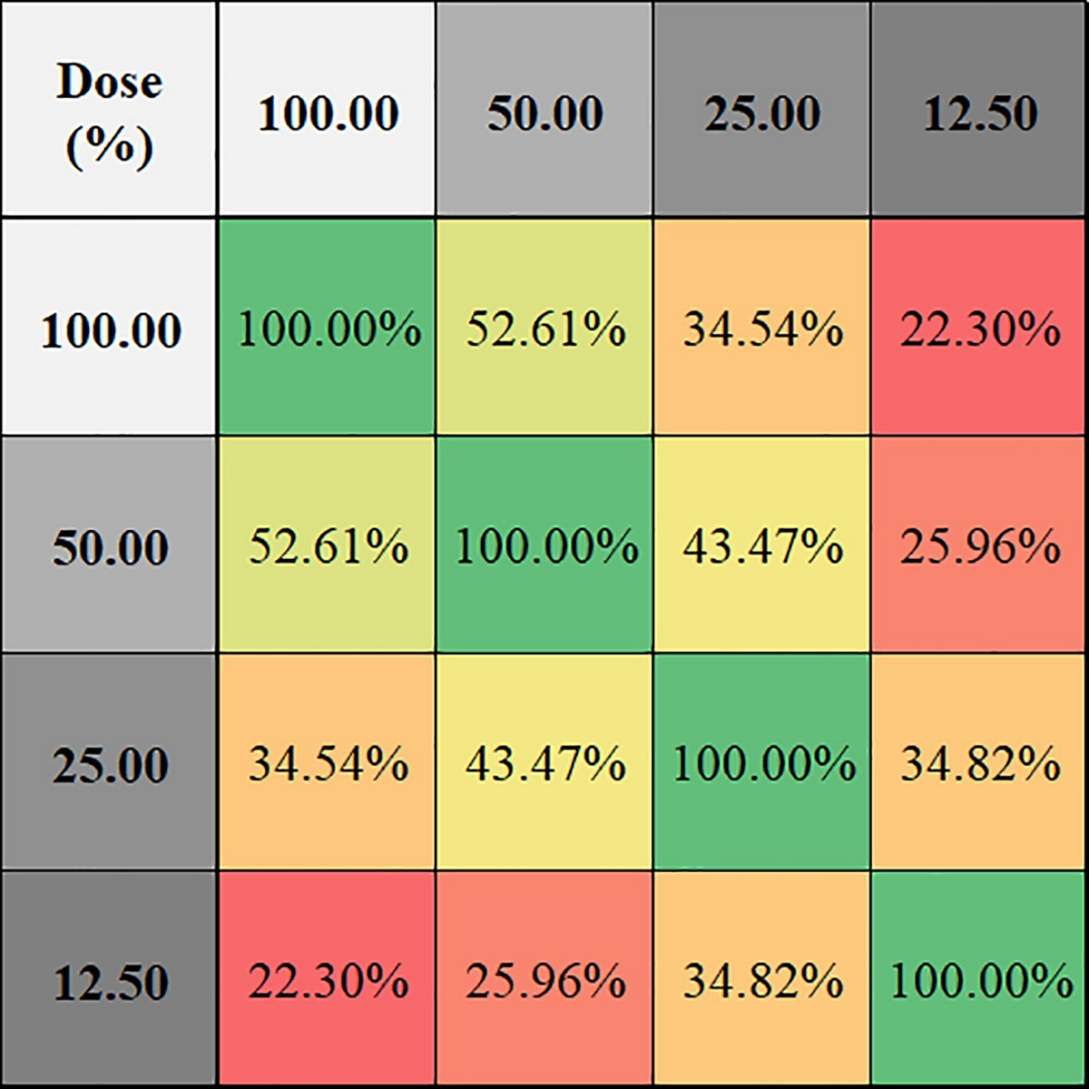
Percentage of compatible texture features in different dose level pairs while keeping kernel and slice thickness fixed for all 23 patients.

320 reconstruction conditions were compared to each other on 28 texture features and 23 cases, totaling 65,945,600 (320x320x28x23) comparisons. As shown in Fig 4, highest average compatibility of 24.47% was achieved using the combination of highest slice thickness, smoothest kernel and highest dose level (5mm/I26f/100%). Compatibility decreases while decreasing slice thickness and/or kernel smoothness and/or dose level. The lowest average compatibility of 2.65% was at the combination of lowest slice thickness, sharpest kernel and lowest dose level (0.6mm/B70f/12.5%). Fig 7 shows percentage compatibilities of radiomic features based on dose, kernel, and thickness changes. The most robust feature was the density against dose changes (87.45% compatibility); and skewness was most robust for kernel and slice-thickness changes (53.73%-82.51% compatibility). Deviation was the weakest feature for all cases. On average, the GLCM based feature group was the most vulnerable feature group (19.41% average compatibility). In addition, results showing percentage compatibilities of conditions based on kernel sharpness, slice thickness, and dose levels are presented in Figs 8, 9 and 10 respectively.

As demonstrated in the figures, while stable volumetric measurements can be obtained (e.g. 2mm slice thickness) and volumetric measurement errors can be predicted when slice thicknesses are changed (Fig 5), that is not the case for radiomic features (Figs 6 and 7). Texture measurements can be very unstable when conditions are altered.

## Discussion

Robust image features are vital for designing and standardizing anatomic and radiomic-based diagnostic and prognostic decision-making [13–17]. In this study, we investigated the effects of image acquisition and reconstruction conditions on volumetric and radiomic features of lung nodules derived from chest CT scans. These conditions contained an extensive list of combinations (320 versions of each examination: 4 doses x 10 kernels x 8 thicknesses).

Lung nodule detection is enhanced when thinner CT slices are produced [18–20]; screening CT scans are preferentially performed with 1 to 2.5-mm-thick slices [18, 21], as was done in the National Lung Screening Trial [22]. Solid nodules ≥ 4-mm in diameter are currently considered important [18] and were the focus of this research.

Accurate and reliable measurement of lung nodule size from CT scans is a key biomarker in the diagnosis of lung cancer. The estimation of nodule growth rates serves as a predictor of malignancy, and size change reflects efficacy of a treatment [23, 24]. A related challenge is the consistency in establishing lung nodule size [18]. Reliable sizing of nodules has traditionally been limited by subjectivity in selection of the desired dimensions to measure and by non-uniformity of manual manipulation of digital calipers; this represents a source of disagreement between interpreters and reference standards [2, 18]. Manually measuring nodule size involves laboriously inspecting all images that include the lesion [23]. To provide a standard method for nodule-size measurement, the RECIST working group proposed the common use of the 1-D maximal diameter as an efficient standard estimator of lesion volume [1, 23, 25]. While such basic mass size measurements are typically used in clinical practice, 3-D volume measurements are growing in importance due to evidence that 3-D volumetry is more robust for quantifying tumor size [24, 26]. Poor agreement between 3-D and simpler methods is commonly seen when the nodule does not conform to spherical or ellipsoidal assumptions that underlie 1-D and 2-D measurements, respectively; in the context of spatial extent for a 3-D object without restrictions on shape, size is best expressed by the volume occupied by the object [23].

Consequently, there is increasing interest in computer-assisted methods aiding the radiologist in measuring the size of lung nodules using volumetric methods [27–30], despite the fact that their calibration and validation becomes a new challenge [23]. Although lung-nodule 3D volumetry has the potential to improve patient management, there is considerable and largely unpredictable variability in its execution related to produced slice thickness, reconstruction algorithm, and scan dose [24, 26, 31, 32]. Our research addressed this issue and demonstrated that while increasing thickness decreases volumetric reproducibility, it improves the reproducibility of histogram- and texture-based features across different acquisition and reconstruction parameters.

Texture analysis is promising for phenotyping and segmenting cancerous tissues [6, 7]. However, Buch et al. [6] highlighted the major looming problem pertaining to radiomics and big data, that despite collecting increasing numbers of radiological images at an exponentially growing rate, the medical field is far from a completely data-driven artificially intelligent diagnosis. The main reason is a lack of data harmony across multi-site studies, which keeps the training data substantially low for a truly large-scale study. Based on the data-mining approach we performed on our results from various parameters, our recommendations are as follows: 1) During volumetric measurements, changes in slice thickness may produce relatively low errors, however the effects on texture are most dramatic. Hence, if possible, slice thickness should not be altered between studies if serial radiomic features are being compared. 2) If scanning/reconstruction changes are inevitable, they should be limited to a single parameter. For example, only dose level or only sharpness should be changed (Figs 8–10), and those changes should be kept to a minimum. Multiple parameter changes, in general, produce greater measurement errors (Fig 4).

We performed a large-scale data mining approach for finding a “compatible” set of parameters, however, as it can be seen from the results, compatibilities are very limited. In an earlier study, Young et al. [24] raised another concern pertaining to radiation dosage and kernel usage in CT lung-nodule volumetry; they found that the volume of lung nodules was extremely robust to the dose and reconstruction kernels. On the other hand, Chen et al. [8, 33–35] showed that the choice of reconstruction algorithm slightly affects the measurement of lesion volume. Regardless, reduced dosage comes at the cost of decreased image quality, which in turn make the results less reproducible for textures. Lo et al. [7] discussed these specific issues pertaining to lung CT and lung nodule quantification. Many authors recognize that it will be desirable to reduce the dose levels among patients, and many vendors are already working towards quantitative imaging based on reduced dose levels [36–39]. In addition, other reports indicated that iterative reconstruction algorithms offer an opportunity to substantially reduce radiation dose in CT scans while maintaining good image resolution for visualization and nodule detections [8, 40–43]. However, the quantitative measurements from iterative algorithms can be significantly different from the traditional FBP algorithms due to varying noise and resolution properties [44]. Novel artificial intelligence-based reconstruction methods may also offer comparable imaging at reduced radiation doses, and these methods should similarly receive careful consideration for quantitative measurements.

Based on earlier studies, high-resolution texture characterization requires image reconstruction using thin slices. However, thin slices also increase image noise; increasing slice thickness will decrease the noise while inducing blur. Based on our results, increasing slice thickness decreases the reproducibility of volumetric measurements but increases the reproducibility of histogram- and texture- based features. Our view based on these results is that there might not be a best universal set of parameters that simultaneously covers both volumetric measures and radiomic features. Increasing slice thickness causes information loss due to smoothing effects [45]. This may be the reason for increased compatibility for texture features.

Our methodology with full control of the reconstruction parameters had advantages and disadvantages. While limiting the number of scanners gave us the full advantage of image reconstruction algorithm compatibility and standardization, our results at this point are limited to few scanner types (e.g. Siemens Definition Flash, Definition AS, AS Plus, Edge) within a single healthcare system. Our data therefore lacked the heterogeneity that exists across CT imaging vendors and providers, and we hope to collaborate with additional institutions in future work. However, the current approach helped us to point out some potential issues in terms of CT image reconstruction.

Another limitation was our relatively small sample size (n = 23). This was due to fact that raw images can only be stored in our scanners for only 2 to 4 weeks (depending on the scanner and due to space restrictions). The study was initially designed as a retrospective analysis on nodule measurements, instead of classification of nodules. Under our circumstances, this would not be possible with retrospective analysis, and we would have needed to conduct a prospective study, which could have taken much longer to complete. Our sample included variations in acquisition parameters, such as tube voltage and tube current, that were not considered independently due to the small sample size. However, these differences were indicative of the heterogeneity in the acquisition parameters of clinical studies at our institution at the time of data collection due to factors such as automatic exposure control and protocol variations between scanners.

Due to the small sample size, we only looked at nodules under 2~2.5 cm for their solid components. If any nodule had surrounding ground glass tissue, these components were ignored (two of our cases had minor ground glass features surrounding them), and only the solid components were included in the measurements and comparisons.

While some texture features are not stable with changing imaging parameters, this does not necessarily mean that a particular feature does not have predictive value within a given algorithm. For a given feature, any recognized instability should be accounted for in algorithm design to limit outcome variance. Future work from our group will consider how feature variations due to imaging parameters affect the outcome of algorithms.

In conclusion, we found that slice thickness is the main factor impacting the reproducibility of the image features we investigated. It is difficult to maintain both volumetric and radiomic measurement reproducibility and reliability simultaneously. However, our findings indicate that at a thickness of approximately 2mm volumetric measurement reproducibility can be maintained. However, especially for reproducibility in radiomic features, image scanning and reconstruction protocols need to remain stable. Standardization of the imaging acquisition parameters would become even more important in larger scale studies, where images are collected from multiple institutions. As we have shown here, even with scanners with compatible image reconstruction parameters in a highly controlled environment, it is hard to maintain measurement reproducibility when parameters are changed. With images coming from multiple sites and multiple vendors, if studies are not designed and scanning protocols are not aligned properly; it can be very hard to produce reliable results that can be utilized within clinical studies.

## Supporting information

S1 Data Collection of numerical data. (XLSX)
